# Rare and low frequency genomic variants impacting neuronal functions modify the Dup7q11.23 phenotype

**DOI:** 10.1186/s13023-020-01648-6

**Published:** 2021-01-06

**Authors:** Farah Qaiser, Yue Yin, Carolyn B. Mervis, Colleen A. Morris, Bonita P. Klein-Tasman, Elaine Tam, Lucy R. Osborne, Ryan K. C. Yuen

**Affiliations:** 1 Department of Molecular Genetics, University of Toronto, Toronto, ON Canada; 2 Genetics & Genome Biology Program, The Hospital for Sick Children, 686 Bay St., Toronto, ON M5G 0A4 Canada; 3 Department of Psychological and Brain Sciences, University of Louisville, Louisville, KY USA; 4 Department of Pediatrics, UNLV School of Medicine, Las Vegas, NV USA; 5 Department of Psychology, University of Wisconsin-Milwaukee, Milwaukee, WI USA; 6 Department of Medicine, University of Toronto, Toronto, ON Canada

**Keywords:** 7q11.23 duplication syndrome, Autism spectrum disorder, Phenotypic variability, Single nucleotide variant, Copy number variant, Whole genome sequencing

## Abstract

**Background:**

7q11.23 duplication (Dup7) is one of the most frequent recurrent copy number variants (CNVs) in individuals with autism spectrum disorder (ASD), but based on gold-standard assessments, only 19% of Dup7 carriers have ASD, suggesting that additional genetic factors are necessary to manifest the ASD phenotype. To assess the contribution of additional genetic variants to the Dup7 phenotype, we conducted whole-genome sequencing analysis of 20 Dup7 carriers: nine with ASD (Dup7-ASD) and 11 without ASD (Dup7-non-ASD).

**Results:**

We identified three rare variants of potential clinical relevance for ASD: a 1q21.1 microdeletion (Dup7-non-ASD) and two deletions which disrupted *IMMP2L* (one Dup7-ASD, one Dup7-non-ASD). There were no significant differences in gene-set or pathway variant burden between the Dup7-ASD and Dup7-non-ASD groups. However, overall intellectual ability negatively correlated with the number of rare loss-of-function variants present in nervous system development and membrane component pathways, and adaptive behaviour standard scores negatively correlated with the number of low-frequency likely-damaging missense variants found in genes expressed in the prenatal human brain. ASD severity positively correlated with the number of low frequency loss-of-function variants impacting genes expressed at low levels in the brain, and genes with a low level of intolerance.

**Conclusions:**

Our study suggests that in the presence of the same pathogenic Dup7 variant, rare and low frequency genetic variants act additively to contribute to components of the overall Dup7 phenotype.

## Background

Duplication of the 7q11.23 region (referred to as Dup7) [1] results in a rare complex neurodevelopmental disorder (MIM 609757) with an estimated prevalence of one in 7500–20,000 individuals [2, 3]. Individuals with a Dup7 copy number variant (CNV) have common Dup7 characteristics including distinctive craniofacial features (i.e., macrocephaly, brachycephaly, broad forehead, straight eyebrows, deep-set eyes, a broad nasal tip with low insertion of the columella, a short philtrum, thin vermillion of the upper lip, minor ear anomalies and facial asymmetry). Structural brain anomalies include ventriculomegaly, decreased white matter volume, and cerebellar vermis hypoplasia, while abnormal neurological findings include hypotonia, abnormalities of gait and station, adventitious movements, seizure disorder, and developmental coordination disorder [4–6]. In addition, common Dup7 characteristics include aortic dilation, developmental delay, low-average intellectual ability, speech sound disorder, social anxiety disorder, selective mutism, and autism spectrum disorder (ASD) [4–6].

Over the past decade, studies have pointed to a strong association between Dup7 and ASD, with Dup7 identified as one of the ten most frequently (0.2%) recurring CNVs found in children with ASD [2, 7–12]. We previously conducted the first systematic characterization of ASD symptomatology in a group of 63 children (aged 4–17 years) with Dup7, finding evidence for an elevated risk of ASD, as 33% of the cohort screened positive for possible ASD [3]. In our subsequent study of an overlapping sample, 19% of the participants with Dup7 received a clinical diagnosis of ASD following gold-standard assessments [4, 5, 9]. Interestingly, in the reciprocal deletion of the 7q11.23 region, which causes Williams-Beuren syndrome (WBS, MIM 194050), the prevalence of ASD symptomatology and diagnosis is also considerably higher than in the general population [13, 14].

Dup7 is a significant ASD risk factor, but despite having the same shared CNV, most individuals with Dup7 do not meet the criteria for ASD diagnosis using a gold-standard diagnostic approach [9]. This phenomenon of incomplete penetrance and phenotypic variability has been observed in a number of neuropsychiatric phenotypes, including those associated with syndromic or multigenic CNVs, and single genes [15–19]. For example, the 22q11 deletion syndrome (22q11DS) is characterized by significant differences in the penetrance of congenital heart defects, schizophrenia and ASD. A recent study found that 20% of 22q11DS carriers with ASD had a second variant within the *mGluR5* network in contrast to only 2% of 22q11DS carriers who did not have ASD, suggesting that a ‘second hit’ significantly contributes to ASD risk in 22q11DS [17]. Similarly, Pizzo et al. [16] assessed the contribution of rare variants in individuals with different primary pathogenic variants, finding that the number of additional hits correlated with the variability and severity of the overall clinical phenotype. Such studies add support to the ‘multiple hit’ model, which proposes that syndromic CNVs exhibit incomplete penetrance and variable expressivity for ASD and instead require additional genetic factors—often referred to as ‘second hits’ or modifiers—in order for individuals to present with ASD [16, 18, 20].

However, there are challenges associated with the multiple hit model, as not all studies have successfully identified additional genetic factors to explain phenotypic variability. For example, Masson et al. [21] carried out whole-exome sequencing (WES) and chromosomal microarray analysis (CMA) for six individuals with WBS-ASD but did not identify any secondary variants which could explain the presence of ASD in these individuals. Such studies highlight the need to utilize methods that allow for a thorough examination of the different variants present in an individual’s genetic background, which will allow for a better understanding of incomplete penetrance and phenotypic variability.

In this study, we carry out the first whole-genome sequencing (WGS) analysis to examine the role of rare (< 1%) and low-frequency (< 5%) variants in the development of ASD in Dup7 carriers. WGS has the potential to identify nearly all forms of genetic variation, including single nucleotide variants (SNVs), indels and CNVs. Previous studies have demonstrated the advantages of WGS for ASD molecular diagnosis, such as a higher molecular diagnostic rate and the ability to detect non-coding variants [22–26]. Since prior studies [15–19, 27] have successfully identified rare secondary variants in individuals with ASD, we carried out rare variant analysis in this Dup7 cohort to identify potential modifiers. By examining the rare and low frequency damaging variants present in each Dup7 carrier’s genetic background, we aimed to better understand how additional variants modulate neuropsychiatric phenotypes in individuals carrying the same primary pathogenic variant.

## Results

### Whole-genome sequencing (WGS) and chromosomal microarray analysis (CMA)

**Fig. 1:**
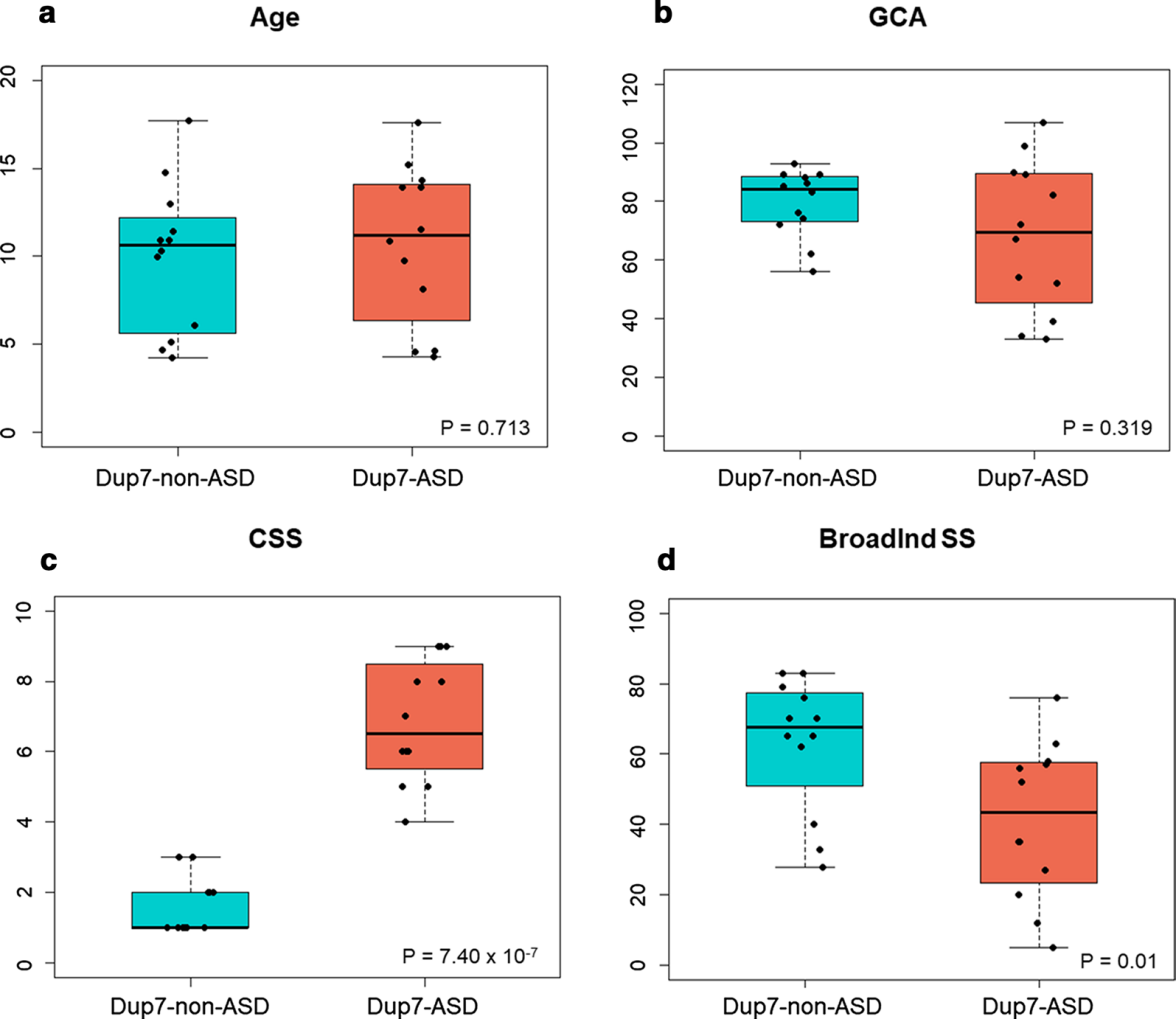
Comparison of demographic and clinical characteristics between the Dup7-ASD and Dup7-non-ASD groups. Boxplots comparing the following clinical characteristics between the Dup7-ASD and the Dup7-non-ASD groups: **a** age (*P* = 0.713); **b** General Conceptual Ability standard score (GCA; similar to IQ) from the Differential Ability Scales-II (*P* = 0.319); **c** Calibrated severity score from the ADOS-2 (*P* = 7.40 × 10^–7^); and **d** Broad Independence standard score from the Scales of Independent Behavior-Revised (*P* = 0.01). Statistically significant differences are marked with an asterisk, where *P* values less than 0.05 are marked with a single asterisk, *P* values less than 0.01 are marked with two asterisks, and *P* values less than 0.001 are marked with three asterisks

Each participant carried a classic Dup7 CNV. For 79.2% of the participants, we were able to determine if the CNV was de novo (17 participants; 71%) or inherited (two participants; 4%; one from each group), and the CNV parent-of-origin. The pattern of CNV parent-of-origin was similar for the two groups: four maternal, five paternal for Dup7-ASD; six maternal, four paternal for Dup7-non-ASD (Fisher exact test, *P* = 0.656). In terms of clinical characteristics, there were no significant differences in sex, age at assessment, or General Conceptual Ability (GCA; similar to IQ) standard scores (SS) between the Dup7-ASD and the Dup7-non-ASD groups (Fig. 1; Additional file 1: Table S3). However, as expected, the Dup7-ASD group had a significantly higher ASD symptom calibrated severity score (CSS) than the Dup7-non-ASD group (*P* = 8.43 × 10^–7^) (Fig. 1; Additional file 1: Table S3). The Dup7-ASD group also had a significantly lower Broad Independence standard score (BroadInd SS, *P* = 0.010), which is a measure of overall adaptive behavior ability, than the Dup7-non-ASD group (Fig. 1; Additional file 1: Table S3).

We sequenced the genomes from 24 Dup7 carriers, with an average of 43.9 × coverage across all samples (Additional file 1: Table S2). One individual had a greater than expected number of rare CNVs and was thus excluded from downstream analyses (Additional file 1: Figure S1, Table S2). When compared to reference samples from the 1000 Genomes project, ancestry analysis found that most of the Dup7 carriers were either European (*n* = 18, 75%) or American (*n* = 3, 12.5%) (Additional file 1: Figure S2, Methods). Of the remaining three Dup7 carriers, one clustered with the South Asian ancestry group, while the other two did not cluster with any group. These three individuals were excluded for potential confounding effects in downstream analyses. Lastly, kinship analysis confirmed that the participants within the cohort were unrelated (Additional file 1: Figure S3).

Overall, a total of nine participants with Dup7-ASD and 11 with Dup7-non-ASD were used in downstream analyses, where we detected an average of 4,685,522 indels and SNVs, and 661 CNVs per genome (Additional file 1: Table S2). Examining rare variants only, we detected an average of 185 indels and SNVs, and 35.4 CNVs impacting coding regions per genome (Additional file 1: Figure S1; Table S3). For the 20 participants used in downstream analyses, there were no significant differences in the distributions of Dup7 CNV size between the Dup7-ASD (Mean: 1,658,333 bp, SD: 175,468 bp) and Dup7-non-ASD (1,564,455 bp, SD: 33,721 bp) groups (Mann–Whitney *U* test, *z* = 0.80, *P* = 0.424) (Additional file 1: Table S4).

### Clinically relevant rare variant analysis

We identified three rare variants of potential clinical relevance to ASD: a pathogenic 1q21.1 microdeletion in an individual with Dup7-non-ASD and two different rare deletions which disrupt *IMMP2L* at the 7q31.1 locus (one Dup7-ASD, one Dup7-non-ASD) (Table 1). These three rare CNVs impact both the coding and non-coding regions of the genome. The latter two CNVs were of different sizes, and both were classified as VUS. We did not identify any SNVs or indels which met clinical significance per American College of Medical Genetics and Genomics (ACMG) guidelines. Overall, there was no significant difference in rare variant findings between the Dup7-ASD group and the Dup7-non-ASD group.

**Table 1:** Rare clinically relevant variants identified in the Dup7 cohort

Group^*^	Gene(s)	Variant type	Variant	Chromosome coordinates (GRCh37/hg19)	Variant category
Dup7-non-ASD	*ACP6, BCL9, CHD1L, FMO5, GJA5, GJA8, GPR89B, NBPF10, NBPF11, NBPF12, NBPF20, NBPF8, PRKAB2*	CNV	DEL	Chr1:146,303,401- 147,891,400 Size: 1,588,000 bp	Pathogenic
Dup7-non-ASD	*IMMP2L, LRRN3*	CNV	DEL	Chr7:110,347,001- 110,883,800 Size: 536,800 bp	Variant of uncertain significance
Dup7-ASD	*IMMP2L*	CNV	DEL	Chr7:111,120,394–111,351,748 Size: 231,355 bp	Variant of uncertain significance

*CNV* copy number variant, *DEL* deletion

^*^Each CNV listed here is found in a different participant

### Burden analysis of genomic variants

To assess the correlation between phenotypic measures and the burden of variants (either rare or low frequency) in participants with Dup7, we performed regression analysis and found that GCA SSs were negatively correlated with the burden of (1) low frequency LoF variants (*P* = 0.012), (2) a combination of low frequency LoF and missense variants (*P* = 0.022), and (3) rare non-Dup7 CNVs (*P* = 0.016) (Additional file 1: Table S5). There was no significant correlation between the burden of genomic variants and the remaining phenotypic measures (i.e. Dup7-ASD vs. Dup7-non-ASD, BroadInd SS, CSS) (Additional file 1: Table S5).

### Correlation of function and pathway with phenotypic outcomes

**Fig. 2:**
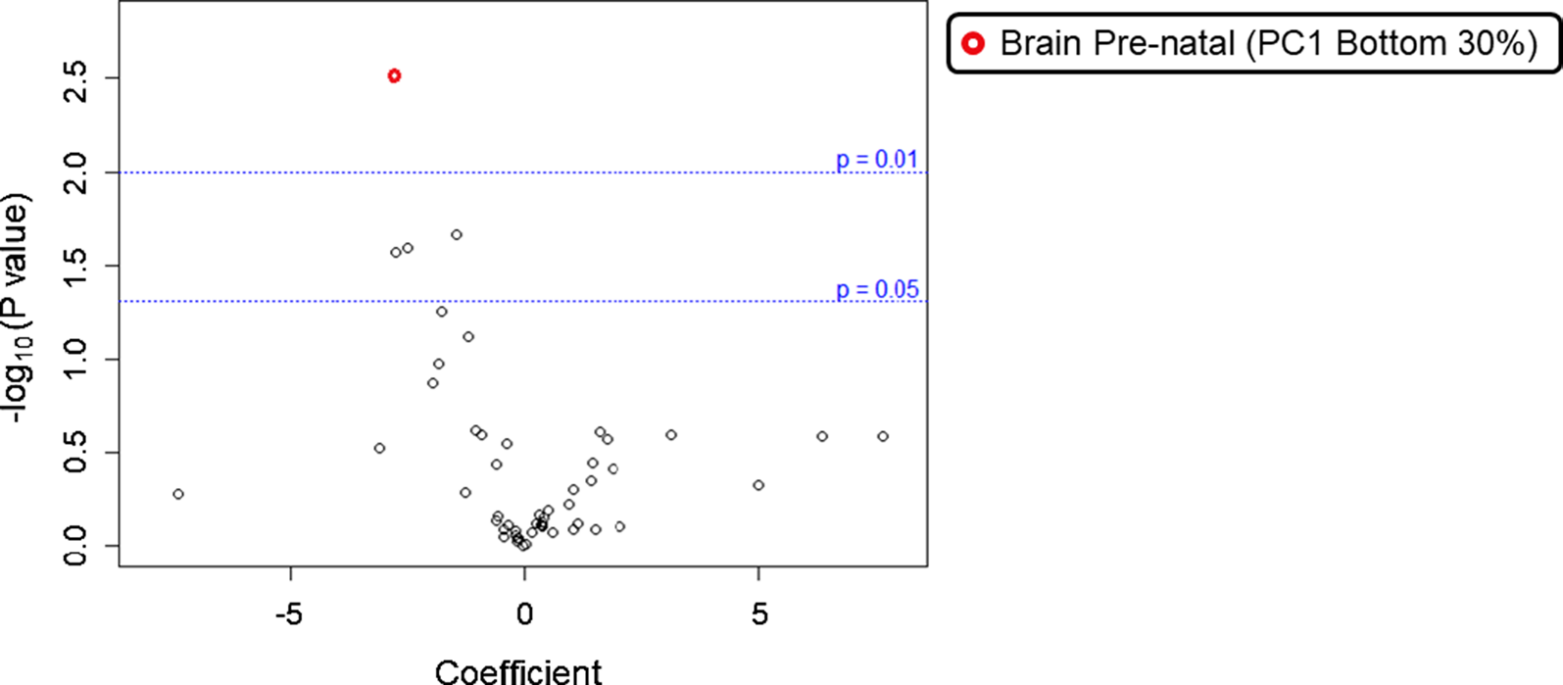
Volcano plot of relationship between BroadInd SS and likely damaging variants present in neuroset pathways. Volcano plot of likely damaging missense variants (5% frequency level) expressed in neuroset pathways for the BroadInd SS outcome variable. Labelled functions met a *P* value cut-off of at most 0.05 and have a false discovery rate (FDR) of ≤ 0.15

**Fig. 3:**
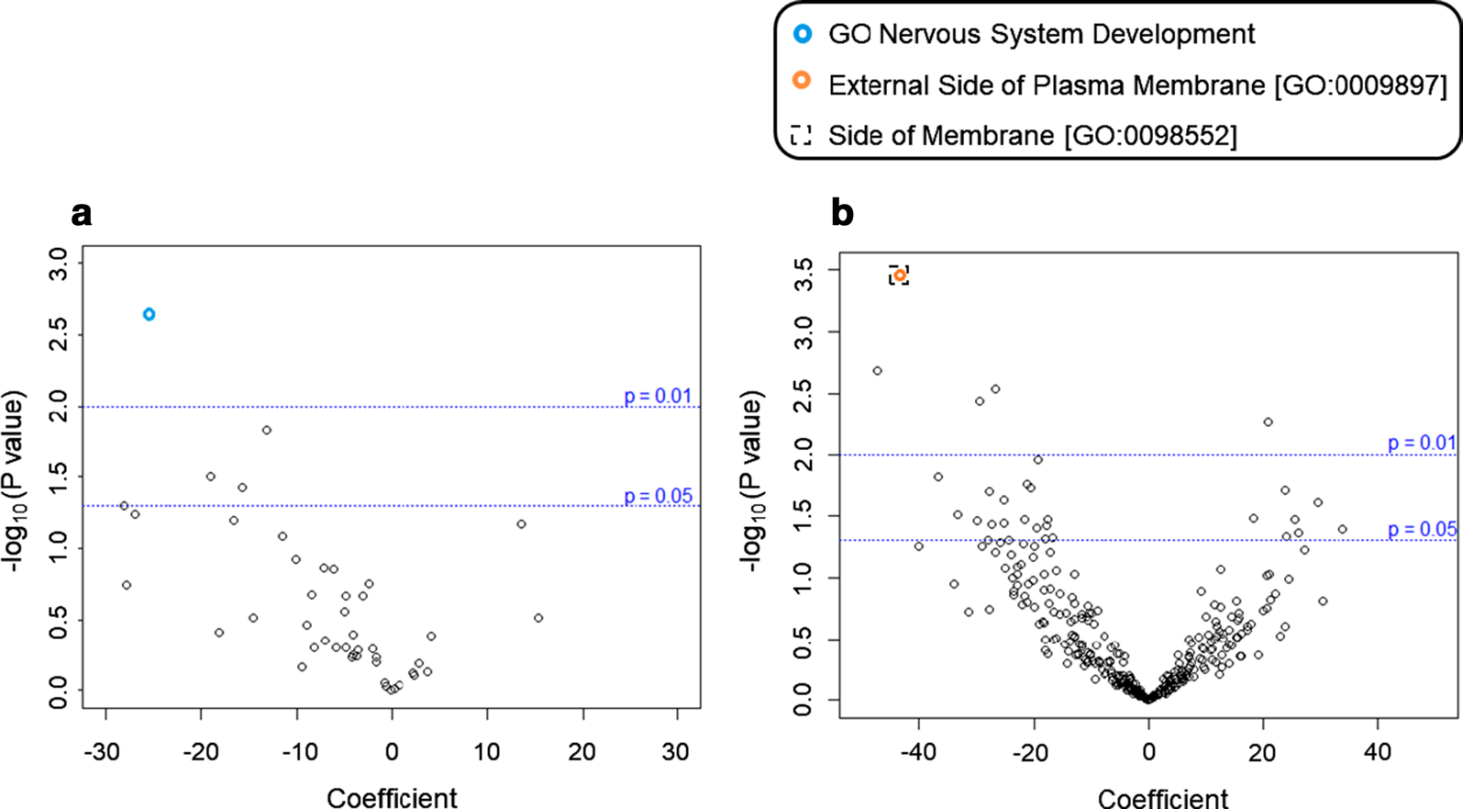
Volcano plot of relationship between GCA and likely damaging variants in GO and neuroset pathways. Volcano plot of rare LoF variants (1% frequency level) expression in neuroset (**a**) and GO (**b**) pathways for the GCA outcome variable. Labelled functions met a *P* value cut-off of at most 0.05 and have a false discovery rate (FDR) of ≤ 0.15. *GCA* DAS-II General Conceptual Ability standard score, *GO* gene ontology, *LoF* loss of function

**Fig. 4:**
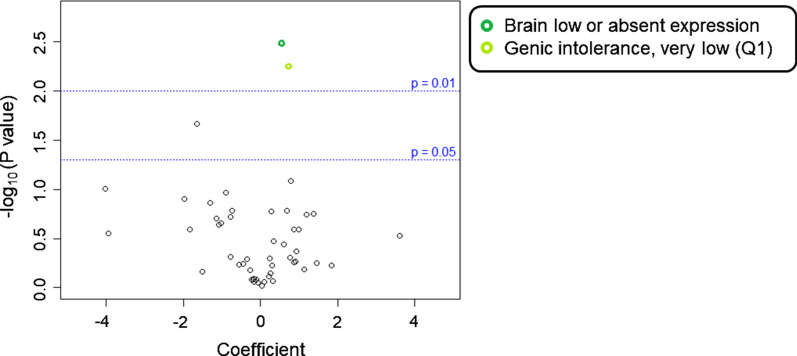
Volcano plot of relationship between CSS and likely damaging variants in GO and neuroset pathways. Volcano plot of low frequency likely damaging LoF (5%) in neuroset pathways for the CSS outcome variable. Labelled functions met a *P* value cut-off of at most 0.05 and have a false discovery rate (FDR) of ≤ 0.15. *CSS* ADOS-2 calibrated severity score, *GO* gene ontology, *LoF* loss of function

To assess the contribution of likely damaging rare and low frequency variants to the overall Dup7 neuropsychiatric phenotype, we conducted regression modelling to identify correlations between phenotypic outcomes and different burden variables in gene-sets (ASD-risk, Neuroset) or pathways (GO, KEGG) (Table 2). Upon examining individual variant types, we found that BroadInd SSs (where higher scores indicate better adaptive behaviour skills) were negatively correlated with likely damaging low frequency missense variants enriched in genes expressed in the prenatal human brain (*P* = 0.003; FDR = 0.078, Fig. 2a). We observed a negative correlation between GCAs (where higher scores indicate higher intellectual ability) and rare likely damaging LoF variants enriched in nervous system development (*P* = 0.002; FDR = 0.074, Fig. 3a), and two membrane component GO pathways (*P* = 3.61 × 10^–4^; FDR = 0.148, Fig. 3B). Lastly, CSSs (where higher scores are associated with higher levels of ASD symptoms) were positively correlated with low frequency LoF variants enriched in genes expressed in low levels in the brain (*P* = 0.003; FDR = 0.078, Fig. 4) and in genes with a very low level of intolerance (*P* = 0.006; FDR = 0.078, Fig. 4).

**Table 2:** Biological processes significantly enriched among genes carrying a higher number of rare or low frequency variants in Dup7 carriers

Outcome phenotype	Variable	Pathway category	Pathway/function	Number of genes in pathway	Number of observed variants	B (coefficient)	*P* value	FDR
BroadInd SS	Likely damaging missense variants (5%)	Neuroset	Brain Pre-natal (PC1 bottom 33%)	3038	451	− 2.75	3.10 × 10^–3^	0.078
CSS	LoF variants (5%)	Neuroset	Brain low/absent expression	4601	145	0.574	3.19 × 10^–3^	0.078
	LoF variants (5%)	Neuroset	Genic intolerance, very low (Q1)	4153	164	0.662	5.87 × 10^–3^	0.078
GCA	LoF variants (1%)	Neuroset	Nervous System Development	1874	8	− 25.2	2.28 × 10^–3^	0.074
	LoF variants (1%)	GO	Side of membrane [GO:0098552]	426	3	− 43.0	3.61 × 10^–4^	0.148
	LoF variants (1%)	GO	External Side of Plasma Membrane [GO:0009897]	232	3	− 43.0	3.61 × 10^–4^	0.148

*CSS* calibrated severity score (from ADOS-2), *GCA* General Conceptual Ability standard score (from DAS-II), *GO* gene ontology, *LoF* loss of function, *PC1* principal component 1, *BroadInd SS* SIB-R Broad Independence standard score. Percentages in parentheses refer to variant frequency

Similarly, to assess the contribution of non-coding variants which cause cryptic splicing to the overall Dup7 neuropsychiatric phenotype, we repeated the above regression modelling analyses, with the inclusion of variants with a high likelihood of disrupting splicing in the LoF SNV burden variable (1%, 5%) (Additional file 1: Table S6). We detected a total of 29 rare and 46 low frequency variants with a high likelihood of disrupting splicing, as predicted by SpliceAI [28]. We found a significant correlation in two of the aforementioned pathways. Specifically, there were significant positive correlations between CSSs and (i) both rare (*P* = 0.004; FDR = 0.091) and low frequency (*P* = 0.002; FDR = 0.036) LoF variants enriched in genes expressed in low levels in the brain, and (ii) low frequency LoF variants impacting genes with a very low level of intolerance (*P* = 0.011; FDR = 0.105).

## Discussion

In this study, we present the first systematic WGS analysis of participants with Dup7, with and without ASD, to explore the role of additional genetic variants in the development of ASD in 7q11.23 duplication syndrome. A comparison of the genetic background between the individuals with Dup7-ASD (*n* = 9) and the individuals with Dup7-non-ASD (*n* = 11) found no significant differences in variant findings between the two groups, but instead showed that in the presence of the same primary pathogenic Dup7 variant, there are additional variants involved, which likely act additively to contribute to the overall intellectual ability and adaptive behavior components of the Dup7 phenotype. These results suggest that in addition to the primary pathogenic variants, rare and low frequency variants present in the genetic background modulate the clinical variability seen in neurodevelopmental disorders (NDDs) such as Dup7.

We identified one pathogenic 1q21.1 deletion and two VUS CNVs of interest impacting *IMMP2L* in this Dup7 cohort, representing 15% of the samples investigated. Haploinsufficiency at the 1q21.1 locus is associated with various phenotypes, and past studies point to 1q21.1 duplications being more strongly associated with ASD or ASD-like features than deletions [29, 30]. This is consistent with the finding of a 1q21.1 deletion in an individual with Dup7-non-ASD in our cohort. We also identified two deletions which disrupted *IMMP2L* and were found in both Dup7-ASD and Dup7-non-ASD participants. Of note, *IMMP2L* encodes the second subunit of the inner mitochondrial membrane peptidase complex and has previously been reported as a potential candidate gene associated with ASD, Tourette syndrome and other NDDs [31–33]. These CNV findings add to the available evidence regarding *IMMP2L*’s potential role in neurodevelopment, but its contribution to ASD remains uncertain given that a CNV disrupting solely *IMMP2L* was identified in a Dup7-non-ASD individual who had an ADOS-2 CSS of 1 (the lowest possible score).

There were no significant differences in Dup7 size, gene-set or pathway variant burden between the Dup7-ASD and Dup7-non-ASD groups. We did identify statistically significant correlations between continuous phenotypic measures (CSS, GCA and BroadInd SS) and rare or low frequency variants in pathways impacting neuronal functions. The implicated pathways include genes found in the Dup7 region, such as *GTF2I, ELN* (the structural protein elastin) and *CLIP2* (a cytoplasmic linker protein), although no likely damaging variants were identified in any of the genes within the Dup7 region, with the exception of *CLIP2*. Of particular interest is the finding that the adaptive behaviour SSs of individuals with Dup7 were negatively correlated with a higher burden of rare likely damaging missense variants in genes expressed in the prenatal human brain. Here, implicated variants included a missense variant in *CLIP2,* and variants in known ASD-risk genes and/or neuropsychiatric phenotypes, such as *SETD5* (a methyltransferase) and *CHD2* (a chromodomain helicase DNA-binding protein). Similarly, we found that overall intellectual ability SSs were negatively correlated with rare likely damaging LoF variants in nervous system development and two membrane GO component pathways, where enriched variants were present in genes encoding receptors (e.g. *CHRNA3*, *AGER*) and cell adhesion molecules (e.g. *PCDHB9*, *PCDHA8*). While some of these enriched variants merit further investigation as they are either present in the Dup7 region or in genes associated with known disorders, it is important to note that none of these variants meet pathogenicity criteria as per ACMG guidelines. Functional characterization of these implicated variants is necessary to understand how they interact with the primary variant (Dup7), other rare and low frequency variants in the genetic background, and how they ultimately modulate the Dup7-associated phenotype.

Taken together, our findings point to a model where rather than discrete second hits, the complexity of clinical characteristics in Dup7-associated neuropsychiatric phenotypes is modulated by rare and low frequency variants, which are present in the genetic background in addition to the primary pathogenic Dup7 variant. This study highlights the power of pathway analyses and the correlation of quantitative traits toward understanding how additional rare variants in the genetic background modulate neurodevelopmental disorders.

Our results should be interpreted in the context of the following limitations. Firstly, we only carried out WGS analysis for the Dup7 carriers in this study, and not parents, thus the inheritance of the identified variants remains unknown. We only examined SNVs and CNVs in the Dup7 cohort, and thus cannot rule out that differences or a burden of other variant types (such as structural variants) may be able to differentiate between the Dup7-ASD and Dup7-non-ASD groups. Finally, it should be noted that because this is a small cohort (*n* = 20), we could not evaluate variants with smaller effect sizes, such as common variants (i.e. ≥ 5% frequency) and non-coding variants, other than those which disrupt splicing. Instead, this study can serve as a baseline when evaluating phenotypic variability in larger Dup7 cohorts.

To date, there has been no analysis of exome or genome data to investigate possible genetic factors in individuals with both Dup7 and ASD, making our study the first to systematically examine the role of rare and low frequency variants in Dup7-ASD. However, exome data have been analyzed for individuals with both ASD and WBS [21, 34]. Codina-Sola et al. (2019) conducted WES in eight individuals with WBS-ASD, where seven (87.5%) of the 7q11.23 deletions were paternal in origin, and a total of five inherited rare variants were identified in ASD-related or loss-of-function intolerant genes, as well as one de novo LoF variant. In contrast, Masson et al. (2019) identified no clinically relevant secondary hits in their WBS-ASD cohort (*n* = 6), and reported 7q11.23 deletions of maternal (*n* = 4) and paternal (*n* = 1) origin in individuals with WBS-ASD. Across the two studies, there is no evidence of an imprinting effect in the WBS-ASD phenotype. Similarly, in our cohort, the pattern of CNV parent-of-origin was similar for both the Dup7-ASD and Dup7-non-ASD groups, providing no evidence of an imprinting effect in the Dup7-ASD phenotype.

As mentioned earlier, the phenomenon of incomplete penetrance and phenotypic variability has been reported in multiple neuropsychiatric phenotypes, suggesting that secondary variants may be necessary for ASD manifestation [15–19, 27]. In our study, we did not identify discrete second hits which could explain the development of ASD in individuals with Dup7, but instead, similar to Pizzo et al. (2018), we identified correlations between different phenotypic measures (such as ASD symptom severity and intellectual ability) and genetic variants enriched in related molecular pathways, suggesting that variants present in the genetic background play a critical role in phenotypic variability.

## Conclusions

Overall, in this study, WGS characterization found that in the presence of the same pathogenic Dup7 variant, additional genetic variants also have an impact on the phenotype. Our findings suggest that neuropsychiatric phenotypes associated with particular syndromes are subject to modulation by additional genetic variants. Thus, assessing the role of rare and low frequency variants present in the genetic background should improve the phenotypic correlation in CNV-associated or monogenic disorders.

## Methods

### Participant cohort

We recruited 24 children with Dup7: 12 individuals with Dup7-ASD (case) and 12 individuals with Dup7-non-ASD (control) who were pairwise matched as closely as possible on sex, age and ethnicity (Additional file 1: Table S1). The presence of classic Dup7 was confirmed in each participant through chromosomal microarray analysis (CMA) using various commercially available platforms and/or quantitative PCR (qPCR). When possible, parents were tested for Dup7, and the parent-of-origin of de novo CNVs was determined by the analysis of single-copy microsatellite markers within the Dup7 region [1]. ASD diagnosis was determined using the gold-standard diagnostic approach [9]. Each participant with Dup7 was clinically evaluated using three standardized measures: the Autism Diagnostic Observation Schedule-2 (ADOS-2) [35], the Differential Ability Scales-II (DAS-II) [36], and the Scales of Independent Behavior-Revised (SIB-R) [37] (see Additional file 1: Methods). Additional file 1: Table S1 reports characteristics for each participant, including sex, ADOS-2 calibrated severity score (CSS, a measure of severity of autism-related symptoms), SIB-R Broad Independence standard score (BroadInd SS, a measure of adaptive behaviour), and DAS-II General Conceptual Ability standard score (GCA, a measure of overall intellectual ability similar to IQ) [38]. Individual ethnicities, Dup7 CNV origins, age at assessment, and the ADOS-2 module administered are not included in order to preserve participants’ privacy. Two-sided non-parametric Mann–Whitney *U* tests were carried out to compare clinical characteristics between the Dup7-ASD and Dup7-non-ASD groups. All procedures were approved by the Research Ethics Boards of the University of Toronto and/or the University of Louisville, and written informed consent was obtained from the parents or legal guardians of all participants.

### Whole genome sequencing (WGS)

Genomic DNA was extracted from blood using the QIAamp DNA Blood Mini Kit (QIAGEN) and then sequenced using the Illumina HiSeq X platform at The Centre for Applied Genomics. Reads were aligned to the reference genome (build GRCh37/hg19) using the Burrows-Wheeler Aligner (v.0.7.12) as a sorted binary alignment map (BAM) format [39]. Duplicate reads were removed by MarkDuplicates from Picard (v.1.133). Local realignment, quality recalibration and removal of duplicate reads were carried out using the Broad Institute’s Genome Analysis Toolkit (GATK) (v.3.4-46) for each genome. Each variant call format (VCF) file was annotated using a custom pipeline based on ANNOVAR, which included annotating effects (such as non-synonymous, nonsense or frameshift variants) and various features (e.g. whether the variant occurs in an exonic, intronic or intergenic region) [40].

To filter for only high-quality variants, the following parameters were applied: (1) autosomal heterozygous variants have a genotype quality (GQ) of ≥ 99, and an alternative allele fraction (AAF) ≥ 0.3 and ≤ 0.7; (2) homozygous variants and those on chromosome X have a GQ ≥ 25 and an AAF > 0.7; and (3) all variants passed GATK pipeline filters. CNVs were detected using a combination of the ERDS and CNVnator read depth based algorithms as previously described [41]. In addition, the SpliceAI tool was used to detect non-coding genetic variants which cause cryptic splicing [28]. To filter for only high-quality variants, the following parameters were applied: (1) at least one of the SpliceAI delta scores (i.e. acceptor or donor gain/loss) ≥ 0.8; and (2) variants which impacted inter-genic regions were excluded.

### Ancestry and Kinship analyses

Ancestry analyses were conducted using the ancestry estimating program ADMIXTURE (v.1.3) and PLINK (v.1.9), where samples from the 1000 Genome Project were used as reference population groups to generate estimated ancestry fractions for each Dup7 carrier (see Additional file 1: Methods) [42]. Through principal component analysis, scatterplots with the top principal components were generated to observe the ancestry clustering pattern of Dup7 carriers. In addition, using PLINK-genome, kinship analysis was carried out to generate estimated identity-by-descent (IBD) proportions to determine the degree of relatedness among the Dup7 carriers.

### Rare variant analysis

A rare variant was defined as one which is present in ≤ 1% of the population in the following databases: the 1000 Genomes project, the Exome Aggregation Consortium, and the Genome Aggregation Database. This includes both missense (non-synonymous) and loss-of-function (LoF) variants, where the latter include frameshift insertions or deletions (indels), nonsense and core splice-site variants. Rare variants in both the coding and non-coding region were prioritized using annotation features such as sequence conservation, biological relevance, probability of LoF intolerance (pLI) scores, genetic mode of inheritance, and their predicted impact on coding and non-coding sequence using in silico algorithms, specifically CADD, SIFT, PolyPhen, Provean, MutationAssessor, MutationTaster, PhyloPMam, and PhyloPVert. Missense variants with high predicted scores in at least four out of eight in silico algorithms were considered likely damaging [24, 26].

As per the ACMG interpretation guidelines, rare CNVs (as called by ERDS and CNVnator from WGS output) were filtered based on their genomic content, and overlap with CNVs reported in databases such as DECIPHER and the Database of Genomic Variants [43, 44]. Rare CNVs were prioritized using various annotation features, including whether the CNV (i) impacted protein coding regions or functionally important elements, (ii) if there was a complete or partial overlap of an established haploinsufficiency genomic region, or (iii) if the reported phenotype was highly specific or consistent with the impacted region, when compared to the literature and/or public databases.

Prioritized rare variants were then classified, as per ACMG guidelines, into the following categories: pathogenic, likely pathogenic, variant of unknown significance (VUS), benign and likely benign [43, 45–47]. Read alignments for variants of interest were manually inspected using Integrative Genomics Viewer. Variants with a high likelihood of disrupting splicing, as predicted by SpliceAI, were not included in this rare variant analysis, as the interpretation guidelines established by the American College of Medical Genetics and Genomics (ACMG) are restricted to the evaluation of genetic variants in canonical ± 1 or 2 splice sites.

### Genomic variant burden analysis

The burden of genomic variants was analyzed using the following regression models in R:

logit(binary outcome variable) = sex + age + burden variable.

continuous outcome variable = sex + age + burden variable.

In each model, the number of qualifying variants per sample at varying frequency levels (either rare [≤ 1%] or low frequency [< 5%]) were defined as the burden variable to test—which included testing (1) LoF SNVs; (2) missense SNVs; and (3) non-Dup7 CNVs impacting both the coding and non-coding regions of the genome. In the case of missense SNVs, all eight prediction algorithms were tested sequentially i.e. mis_1 refers to one of the eight algorithms supporting that a missense variant was likely damaging, mis_2 refers to two supporting algorithms, etc.

Both binary (i.e. Dup7-ASD or Dup7-non-ASD) and continuous (SIB-R and DAS-II SSs) phenotypic measures were used as outcome variables to construct models for correlation studies. Each model also included the participant’s sex and age as covariates for potential confounding effects. Control models were constructed using synonymous variants and non-frameshift indels to see the potential effect of other confounders. The false discovery rate (FDR) was calculated to account for multiple comparisons.

For each burden analysis, the target variable had to meet the following thresholds to reach significance: (1) the B (raw coefficient) was positive for models using Dup7-ASD/Dup7-non-ASD or CSS as an outcome variable and negative for models using GCA and BroadInd SS as an outcome variable; (2) met a *P* value cut-off of at most 0.05; and (3) had an FDR value ≤ 0.15. One-tailed statistics are reported, and corresponding volcano plots were generated for each model where the target variable met significance.

### Function and pathway enrichment analyses

Similar to the above burden analyses, function and pathway enrichment analyses on gene-sets with rare and low frequency variants were carried out on the Dup7 cohort using regression models in R. Here, we correlated the results with categorical and quantitative phenotypic measures (i.e. Dup7-ASD or Dup7-non-ASD, or SIB-R and DAS-II SSs). We tested the following burden variables: (1) LoF SNVs; (2) likely damaging missense SNVs (as defined above i.e. at least four out of eight in silico algorithms supported the variant’s likely damaging effect); and (3) non-Dup7 CNVs. Two different gene-sets were used: (1) a combination of an ASD-related gene list (consisting of 1132 genes that have been reported to have an association with ASD) and 51 neuro-related gene-sets i.e., Neuroset (which were collected based on their neural function, brain expression or neural disease phenotypes); and (2) two commonly used gene pathway systems: Geno Ontology (GO) and the Kyoto Encyclopedia of Genes and Genomes (KEGG), where the gene-set size was limited to 100–1000 genes [24, 26, 48].

The function and pathway analyses were also repeated to include non-coding genetic variants with a high likelihood of disrupting splicing, as predicted by SpliceAI, in the LoF SNV burden variable (1%, 5%).

## Supplementary Information

**Additional file 1**. Supplementary Materials and Methods.

## Data Availability

The dataset(s) supporting the conclusions of this article is(are) included within the article (and its supplementary information files). Individual ethnicities, Dup7 CNV origins, age at assessment, and the ADOS-2 module administered are not included in order to preserve participants’ privacy.

## Abbreviations

AAF: Alternative allele fraction
ACMG: American College of Medical Genetics and Genomics
ADOS-2: Autism Diagnostic Observation Schedule-2
ASD: Autism spectrum disorder
BroadInd SS: SIB-R Broad Independence standard score
CMA: Chromosomal microarray analysis
CNV: Copy number variant
CSS: Calibrated severity score
DAS-II: Differential Ability Scales-II
DEL: Deletion
Dup7: Duplication of the 7q11.23 region
FDR: False discovery rate
GATK: Genome analysis toolkit
GCA: DAS-II General Conceptual Ability standard score
GO: Geno ontology
GQ: Genotype quality
IBD: Identity-by-descent
Indel: Insertion or deletion
KEGG: Kyoto Encyclopedia of Genes and Genomes
LoF: Loss-of-function
NDD: Neurodevelopmental disorder
P: *P* Value
PC: Principal component
pLI: Probability of LoF intolerance
qPCR: Quantitative polymerase chain reaction
SIB-R: Scales of Independent Behavior-Revised
SNV: Single nucleotide variant
VCF: Variant call format
VUS: Variant of uncertain significance
WBS: Williams-Beuren Syndrome
WES: Whole-exome sequencing
WGS: Whole-genome sequencing
22q11DS: 22Q11 deletion syndrome
