# Supplementary material for: Rare and low frequency genomic variants impacting neuronal functions modify the Dup7q11.23 phenotype
Source: Orphanet J Rare Dis. 2021 Jan 6;16:6. doi: 10.1186/s13023-020-01648-6 (PMC7788915; doi:10.1186/s13023-020-01648-6)

**Additional file 1**

**Materials and Methods**

**Participant Cohort**

The participants with Dup7 were assessed using the following standardized measures:

1. *Autism Diagnostic Observation Schedule-2 (ADOS-2)*

The ADOS-2 (1) is a structured, play-like interaction with a trained examiner designed to assess ASD-related impairments in communication and reciprocal social interaction, play and imagination, and the presence of restricted and repetitive behaviours and interests (2). It involves structured materials, tasks and observations. The examiner selects the ADOS module that best matches the child’s expressive language skills. Module 1 is designed for children who do not have expressive spoken language or speak primarily in single-word utterances, Module 2 for children and adolescents who produce spontaneous 3-word phrases sometimes including a verb but who are not fluent, and Module 3 for children and adolescents who are verbally fluent.

A subset of the items scored makes up the “ADOS-2 Algorithm,” empirically derived to distinguish between children with and without ASD. The ADOS-2 algorithm yields item scores, an algorithm raw summary score, and an algorithm classification (1 = autism, 2 = ASD, and 3 = non-spectrum) based on empirically derived cut-offs. The ADOS-2 also yields a calibrated severity score (CSS) (3), which ranges from 1 to 10, to describe the level of the child’s ASD-related symptoms compared to those of children with known ASD diagnoses of about the same chronological age and language level. Scores of 1–2 indicate minimal-to-no evidence of autism spectrum-related symptoms, 3–4 indicate low evidence, 5–7 indicate moderate evidence, and 8–10 indicate high evidence of autism spectrum-related symptoms.

1. *Differential Ability Scales-II (DAS-II)*

The DAS-II Early Years (for ages 4–8 years) and DAS-II School Age (for ages 9–17 years) were used to assess a range of intellectual abilities in the Dup7 (4). The DAS-II provides a General Conceptual Ability standard score (GCA; similar to IQ) which is based on the child’s performance on the six core subtests, which measure verbal ability, nonverbal reasoning ability and spatial ability, relative to same-aged children in the national norming sample. For the general population, mean GCA = 100 (SD = 15, range: 30–170), with higher scores indicating higher overall intellectual ability.

1. *Scales of Independent Behavior-Revised (SIB-R)*

The SIB-R parent interview form (5) was used to measure the child’s adaptive behaviour – the ability to function independently at home, at school, and in the community. Four clusters of adaptive skills are included: Motor Skills, Social Interaction and Communication Skills, Personal Living Skills, and Community Living Skills. The overall Broad Independence standard score (BroadInd SS) is based on the child’s performance on all four clusters relative to same-aged children in the normative sample. For the general population, mean BroadInd SS = 100 (SD = 15, range: 1–180), with higher scores indicating greater ability to function independently.

**Ancestry & Kinship Analyses**

To run the ancestry analysis, the original participant VCF files were first pruned by the --indep-pairwise option of PLINK to adjust for linkage disequilibrium. Next, the pruned binary data (.bed) from PLINK, and the optimal K value (determined by ADMIXTURE’s cross-validation procedure), were used as input for ADMIXTURE’s ancestry estimation. The program output is a *.Q file which lists the estimated ancestry fractions for each individual. Using R, principal component analysis (PCA) was further conducted on the ancestry fractions. Lastly, scatter plots of the top principal components (PCs) were generated to observe the clustering pattern of samples with reference population groups from the 1,000 Genome project, which are as follows: Africans, Americans, East Asians, Europeans and South Asians (6). Here, Americans in the reference population group include Colombians, individuals of Mexican ancestry living in California, Peruvians and Puerto Ricans (6).

A kinship analysis was carried out, using the --genome option of PLINK, to determine the degree of relatedness among samples and to discover any anomalies, such as unknown familial relationships, sample contamination or pedigree errors (7). The input PLINK binary file (.bed) consisted of 1,989,184 high quality SNPs from the 1000 Genome Project, which were further reduced using the --geno 0.01 option. The output *.genome file contained the estimated identity-by-descent (IBD) proportions for pairwise individuals, where IBD values less than 0.1 indicate an independent relationship.

**References**

1. Lord C, Rutter M, DiLavore P, Risi S, Gotham K, Bishop S. Autism Diagnostic Observation Schedule, 2nd edition. Los Angeles, CA: Western Psychological Services; 2012.

2. Lord C, Rutter M, Goode S, Heemsbergen J, Jordan H, Mawhood L, et al. Autism Diagnostic Observation Schedule: A standardized observation of communicative and social behavior. J Autism Dev Disord. 1989;19(2):185–212.

3. Gotham K, Pickles A, Lord C. Standardizing ADOS scores for a measure of severity in autism spectrum disorders. J Autism Dev Disord. 2009;39(5):693–705.

4. Elliott C. Differential Ability Scales, 2nd edition. San Antonio: TX: Psychological Corporation; 2007.

5. Bruininks R, Woodcock R, Weatherman R, Hill B. Scales of Independent Behavior – Revised. Chicago: IL: Riverside; 1996.

6. Roslin NM, Li W, Paterson AD, Strug LJ. Quality control analysis of the 1000 Genomes Project Omni2.5 genotypes. bioRxiv. 2016.

7. Stevens EL, Heckenberg G, Roberson EDO, Baugher JD, Downey TJ, Pevsner J. Inference of relationships in population data using identity-by-descent and identity-by-state. PLoS Genet. 2011;7(9).

**Table S1: Demographic and clinical characteristics of participants with Dup7**

| **Participant number** | **Sex** | **ASD Diagnosis** | **ADOS-2 CSS** | **GCA** | **BroadInd SS** |
| --- | --- | --- | --- | --- | --- |
| 2628 | M | No | 1 | 62 | 40 |
| 4126 | M | No | 1 | 76 | 28 |
| 4138 | M | Yes | 6 | 72 | 57 |
| 4284 | M | Yes | 6 | 33 | 5 |
| 4302 | F | Yes | 4 | 52 | 12 |
| 4309 | M | No | 1 | 93 | 76 |
| 4324 | M | Yes | 5 | 89 | 52 |
| 4328 | M | No | 1 | 89 | 83 |
| 4407 | M | No | 1 | 88 | 70 |
| 4424 | F | No | 3 | 72 | 70 |
| 4434 | M | Yes | 9 | 67 | 56 |
| 4442 | F | Yes | 5 | 107 | 63 |
| 4459 | M | No | 2 | 85 | 65 |
| 4463 | M | Yes | 6 | 39 | 35 |
| 4481 | M | No | 2 | 74 | 79 |
| 4504 | F | No | 3 | 83 | 83 |
| 4517 | M | No | 1 | 56 | 62 |
| 4525 | M | Yes | 9 | 90 | 20 |
| 4536 | M | Yes | 9 | 34 | 35 |
| 4583 | F | No | 1 | 86 | 65 |
| 4608 | M | Yes | 7 | 99 | 76 |
| 4662 | M | No | 2 | 89 | 33 |
| 4700 | M | Yes | 8 | 82 | 58 |
| 4718 | M | Yes | 8 | 54 | 27 |

ADOS-2 CSS, Autism Diagnostic Observation Schedule-2 calibrated severity score; GCA, Differential Ability Scales-II General Conceptual Ability standard score; BroadInd SS, Scales of Independent Behavior-Revised Broad Independence standard score.

**Table S2: Summary of Dup7 cohort genome sequencing metrics**

| **Sample** | **Coverage** | **Total indels  and SNVs** | **Rare indels**  **and SNVs** | **Total CNVs** | **Rare CNVs** |
| --- | --- | --- | --- | --- | --- |
| 2628 | 34.93 | 4586568 | 205 | 342 | 34 |
| 4126 | 39.39 | 4607159 | 177 | 656 | 22 |
| 4138 | 41.75 | 4663707 | 186 | 520 | 37 |
| 4284 | 41.37 | 4690147 | 133 | 112 | 83 |
| 4302 | 41 | 4742212 | 176 | 558 | 32 |
| 4309 | 52.44 | 4659799 | 199 | 775 | 37 |
| 4324 | 43.98 | 4632439 | 166 | 741 | 26 |
| 4328 | 44.96 | 4596378 | 184 | 734 | 30 |
| 4407 | 48.1 | 4627846 | 189 | 765 | 35 |
| 4424 | 46.94 | 4810418 | 188 | 757 | 44 |
| 4434 | 47.07 | 4668033 | 182 | 759 | 26 |
| 4442 | 47.97 | 4722764 | 187 | 552 | 175 |
| 4459 | 45.09 | 4656273 | 187 | 729 | 30 |
| 4463 | 44.11 | 5003322 | 236 | 745 | 62 |
| 4481 | 46.95 | 4663943 | 152 | 657 | 23 |
| 4504 | 42.76 | 4662236 | 188 | 558 | 29 |
| 4517 | 46.62 | 4647759 | 172 | 779 | 41 |
| 4525 | 42.94 | 4617577 | 168 | 450 | 23 |
| 4536 | 43.37 | 4839072 | 200 | 748 | 56 |
| 4583 | 45.13 | 4698581 | 184 | 724 | 32 |
| 4608 | 39.51 | 4653237 | 190 | 623 | 35 |
| 4662 | 38.18 | 4625745 | 174 | 601 | 50 |
| 4700 | 42.26 | 4733792 | 191 | 654 | 47 |
| 4718 | 49.65 | 4643534 | 174 | 756 | 48 |

CNV, copy number variant; SNV, single nucleotide variant; WGS, whole genome sequencing

**Table S3: Comparison of demographic and clinical characteristics between the Dup7-non-ASD and Dup7-ASD groups**

|  | **Dup7-non-ASD mean (SD)** | **Dup7-ASD  mean (SD)** | **Test (z-value)** | **Exact  P-value** |
| --- | --- | --- | --- | --- |
| **Sex** | 9 males, 3 females | 10 males, 2 females | 2-sided Fisher exact | 1.00 |
| **Age** | 9.88 (4.12) | 10.7 (4.51) | 2-sided Mann-Whitney U  (z = 0.40) | 0.713 |
| **CSS** | 1.58 (0.79) | 6.83 (1.75) | 2-sided Mann-Whitney U (z = 4.22) | **7.40 x 10^-7^** |
| **GCA** | 79.4 (11.6) | 68.2 (25.6) | 2-sided Mann-Whitney U (z = -1.01) | 0.319 |
| **BroadInd SS** | 62.8 (19.0) | 41.3 (22.3) | 2-sided Mann-Whitney U (z = -2.51) | **0.010** |

Statistically significant values are shown in bold.

CSS, ADOS-2 calibrated severity score; GCA, Differential Ability Scales-II General Conceptual Ability standard score; BroadInd SS, Scales of Independent Behavior-Revised Broad Independence standard score.

**Table S4: Summary of cohort Dup7 size (in bp)**

| **Participant ID** | **Group** | **WGS Chr7 Coordinates** | | | | | | **Size** |
| --- | --- | --- | --- | --- | --- | --- | --- | --- |
|  |  | **Call 1** | **Call 1** | **Call 2** | **Call 2** | **Call 3** | **Call 3** |  |
| 2628 | Dup7-non-ASD | 72589001 | 73829000 | 73831001 | 74241000 |  |  | 1,650,000 |
| 4126 | Dup7-non-ASD | 72603001 | 73829000 | 73831001 | 74190000 |  |  | 1,585,000 |
| 4138 | Dup7-ASD | 72588001 | 73550000 | 73551001 | 74634000 |  |  | 2,045,000 |
| 4302 | Dup7-ASD | 72589001 | 74342000 |  |  |  |  | 1,753,000 |
| 4309 | Dup7-non-ASD | 72642001 | 73829000 | 73831001 | 74172000 |  |  | 1,528,000 |
| 4324 | Dup7-ASD | 72646001 | 73829000 | 73831001 | 74202000 |  |  | 1,554,000 |
| 4328 | Dup7-non-ASD | 72646001 | 73829000 | 73831001 | 74209000 |  |  | 1,561,000 |
| 4407 | Dup7-non-ASD | 72628001 | 73550000 | 73551001 | 73829000 | 73831001 | 74166000 | 1,535,000 |
| 4434 | Dup7-ASD | 72626001 | 74183000 |  |  |  |  | 1,557,000 |
| 4459 | Dup7-non-ASD | 72641001 | 73829000 | 73831001 | 74213000 |  |  | 1,570,000 |
| 4463 | Dup7-ASD | 72629001 | 73550000 | 73551001 | 73828000 | 73831001 | 74183000 | 1,550,000 |
| 4481 | Dup7-non-ASD | 72602001 | 74183000 |  |  |  |  | 1,581,000 |
| 4504 | Dup7-non-ASD | 72638001 | 73828000 | 73831001 | 74195000 |  |  | 1,554,000 |
| 4517 | Dup7-non-ASD | 72681001 | 73550000 | 73551001 | 74218000 |  |  | 1,536,000 |
| 4525 | Dup7-ASD | 72647001 | 74215000 |  |  |  |  | 1,568,000 |
| 4536 | Dup7-ASD | 72629001 | 73829000 | 73831001 | 74190000 |  |  | 1,558,000 |
| 4583 | Dup7-non-ASD | 72639001 | 73829000 | 73831001 | 74195000 |  |  | 1,554,000 |
| 4608 | Dup7-ASD | 72686001 | 74221000 |  |  |  |  | 1,535,000 |
| 4662 | Dup7-non-ASD | 72647001 | 74202000 |  |  |  |  | 1,555,000 |
| 4718 | Dup7-ASD | 72588001 | 73829000 | 73831001 | 74395000 |  |  | 1,805,000 |

**Table S5: Summary of burden analysis of genomic variants**

| **Model** | logit(Dup7-ASD) = Sex + Age + burden variable | | ADOS-2 CSS = Sex + Age + burden variable | | DAS-II GCA = Sex + Age + burden variable | | SIBR BroadInd SS = Sex + Age + burden variable | |
| --- | --- | --- | --- | --- | --- | --- | --- | --- |
| **Burden variable  (1% or 5%)** | **B  Coefficient** | **P-value** | **B Coefficient** | **P-value** | **B Coefficient** | **P-value** | **B Coefficient** | **P-value** |
| lof_mis_0.05 | 0.018 | 0.138 | 0.020 | 0.085 | **-0.157** | **0.022** | -0.073 | 0.419 |
| lof_0.05 | 0.213 | 0.135 | 0.339 | 0.073 | **-2.740** | **0.012** | -1.969 | 0.170 |
| mis_0.05 | 0.019 | 0.144 | 0.021 | 0.092 | **-0.161** | **0.026** | -0.072 | 0.448 |
| mis_1_0.05 | 0.019 | 0.150 | 0.021 | 0.100 | **-0.168** | **0.025** | -0.069 | 0.488 |
| mis_2_0.05 | 0.016 | 0.277 | 0.023 | 0.280 | **-0.242** | **0.045** | -0.112 | 0.476 |
| mis_3_0.05 | 0.017 | 0.376 | 0.027 | 0.362 | -0.322 | 0.066 | -0.160 | 0.476 |
| mis_4_0.05 | 0.001 | 0.975 | 0.006 | 0.873 | -0.373 | 0.101 | -0.345 | 0.225 |
| mis_5_0.05 | 0.005 | 0.889 | 0.021 | 0.697 | -0.502 | 0.103 | -0.358 | 0.357 |
| mis_6_0.05 | 0.008 | 0.877 | 0.035 | 0.665 | -0.667 | 0.155 | -0.152 | 0.799 |
| mis_7_0.05 | 0.036 | 0.579 | 0.084 | 0.411 | -1.093 | 0.064 | -0.665 | 0.380 |
| mis_8_0.05 | -0.121 | 0.332 | -0.055 | 0.779 | 0.423 | 0.718 | 0.286 | 0.843 |
| lof_mis_0.01 | 0.025 | 0.431 | 0.036 | 0.477 | -0.336 | 0.259 | -0.006 | 0.987 |
| lof_0.01 | 0.317 | 0.153 | 0.294 | 0.301 | -1.706 | 0.321 | -1.382 | 0.516 |
| mis_0.01 | 0.016 | 0.597 | 0.026 | 0.599 | -0.280 | 0.347 | 0.036 | 0.924 |
| mis_1_0.01 | 0.018 | 0.545 | 0.026 | 0.599 | -0.322 | 0.271 | 0.022 | 0.952 |
| mis_2_0.01 | 0.012 | 0.758 | 0.010 | 0.878 | -0.355 | 0.371 | 0.105 | 0.831 |
| mis_3_0.01 | 0.006 | 0.913 | 0.002 | 0.978 | -0.357 | 0.493 | 0.450 | 0.482 |
| mis_4_0.01 | -0.030 | 0.608 | -0.054 | 0.558 | -0.438 | 0.431 | -0.204 | 0.767 |
| mis_5_0.01 | -0.041 | 0.586 | -0.040 | 0.743 | -0.663 | 0.363 | 0.319 | 0.725 |
| mis_6_0.01 | 0.008 | 0.936 | 0.050 | 0.757 | -1.013 | 0.291 | 0.565 | 0.636 |
| mis_7_0.01 | 0.047 | 0.650 | 0.085 | 0.608 | -1.753 | 0.064 | -0.251 | 0.838 |
| mis_8_0.01 | 0.024 | 0.906 | 0.182 | 0.582 | -0.678 | 0.735 | -0.715 | 0.771 |
| non_dup7_cnv_0.05 | 0.062 | 0.131 | 0.090 | 0.105 | -0.585 | 0.077 | -0.084 | 0.843 |
| non_dup7_cnv_0.01 | 0.085 | 0.148 | 0.122 | 0.124 | **-1.098** | **0.016** | -0.735 | 0.218 |

Statistically significant P-values (P ≤ 0.05) are shown in bold.

Lof, loss-of-function; mis, missense; mis_X, missense where X refers to the number of algorithms which classify the missense variant as likely damaging.

**Table S6:** **Biological processes significantly enriched among genes carrying a higher number of rare or low frequency variants (with the inclusion of variants with a high likelihood of disrupting splicing) in Dup7 carriers.**

| Outcome Phenotype | Variable | Pathway Category | Pathway/  Function | Number of Genes in Pathway | Number of Observed Variants | B (Coefficient) | P-value | FDR |
| --- | --- | --- | --- | --- | --- | --- | --- | --- |
| CSS | LoF variants (1%) | Neuroset | Brain low/absent expression | 4601 | 81 | 0.960 | 3.79 x 10^-3^ | 0.091 |
|  | LoF variants (5%) | Neuroset | Brain low/absent expression | 4601 | 155 | 0.560 | 1.89 x 10^-3^ | 0.036 |
|  | LoF variants (5%) | Neuroset | Genic intolerance, very low (Q1) | 4153 | 175 | 0.565 | 1.10 x 10^-2^ | 0.105 |

CSS, calibrated severity score (from ADOS-2); LoF, loss of function. Percentages in parentheses refer to variant frequency.

**Figure Legends**

**Figure S1: Aggregate variant counts for the Dup7 cohort.** A plot of aggregate variant counts, where **A** displays single nucleotide variants (at 1% and 5% frequency), and **B** displays rare exonic copy number variants (also at 1% and 5% frequency). There was one CNV outlier (4442). LoF, loss-of-function; mis, missense.

**Figure S2: Scatterplot depicting a principal component analysis of the Dup7 cohort.**This scatterplot depicts the top two principal components (PCs) from a principal component analysis, where 1000 Genome Project samples were used as reference population groups to generate ancestry clusters for each Dup7 carrier (n=24). There were two participants who failed to cluster with the reference ethnic groups.

**Figure S3: Kinship analysis of the Dup7 cohort.** A plot of pairwise identity-by-descent (IBD) proportions to determine the degree of relatedness among samples. An IBD proportion of 0.10 was set as the threshold for an independent relationship.

**Figure S1**


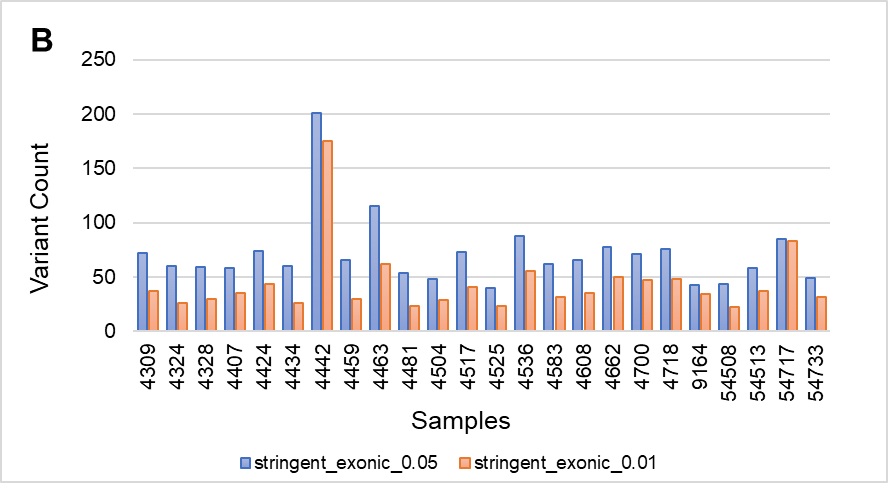

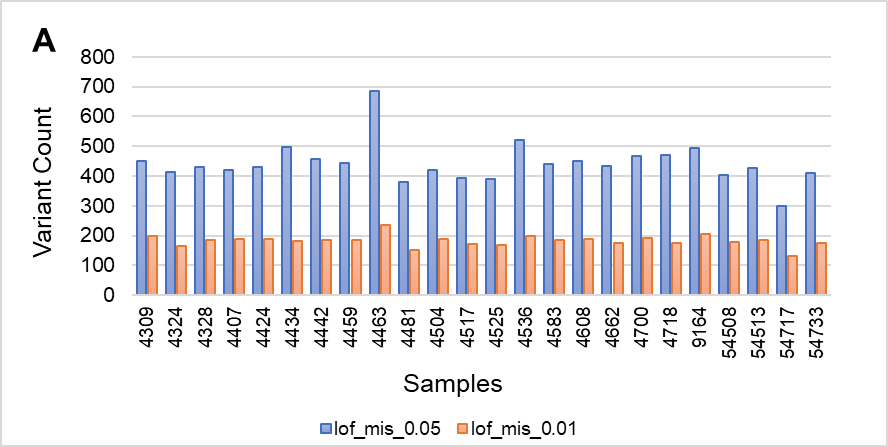


**Figure S2**


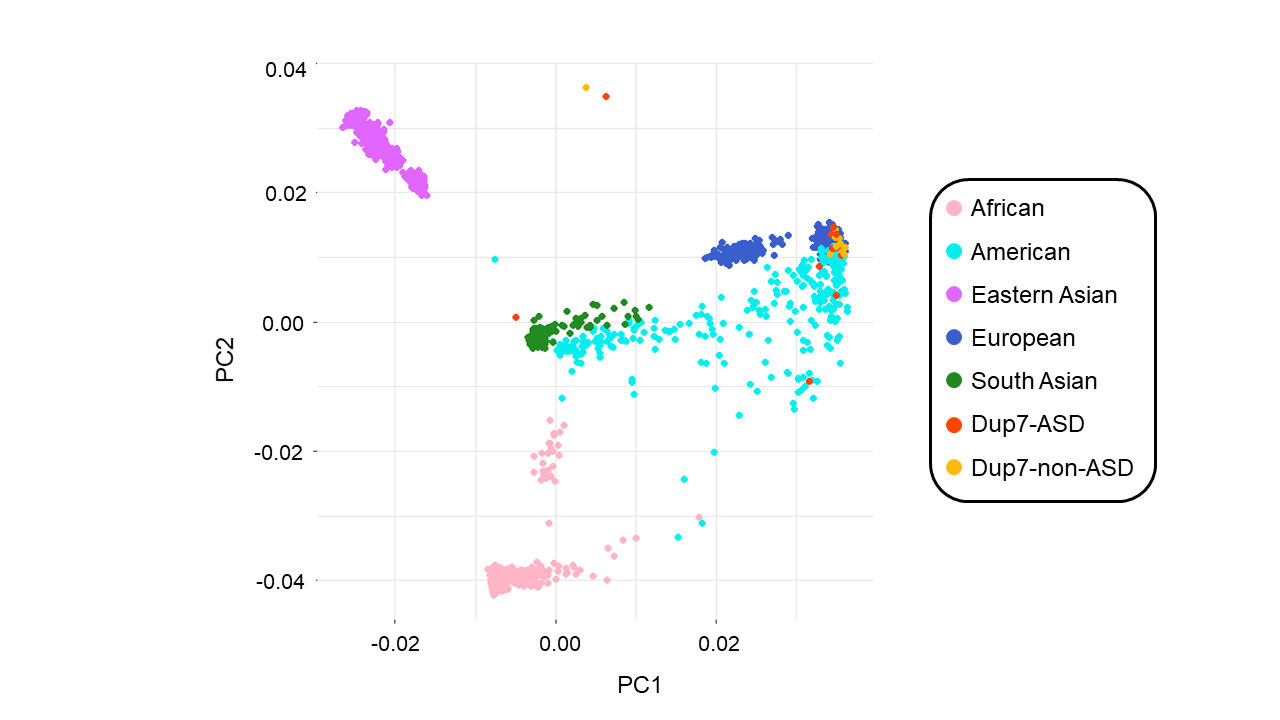

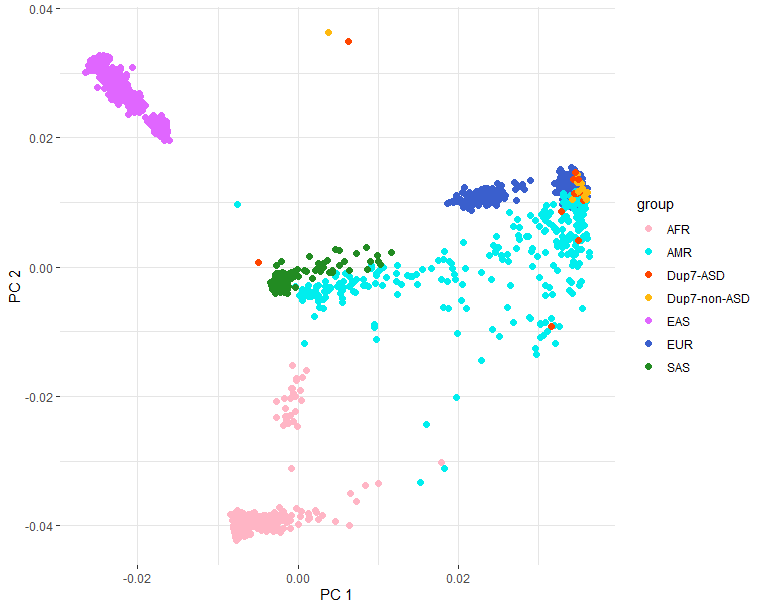


**Figure S3**


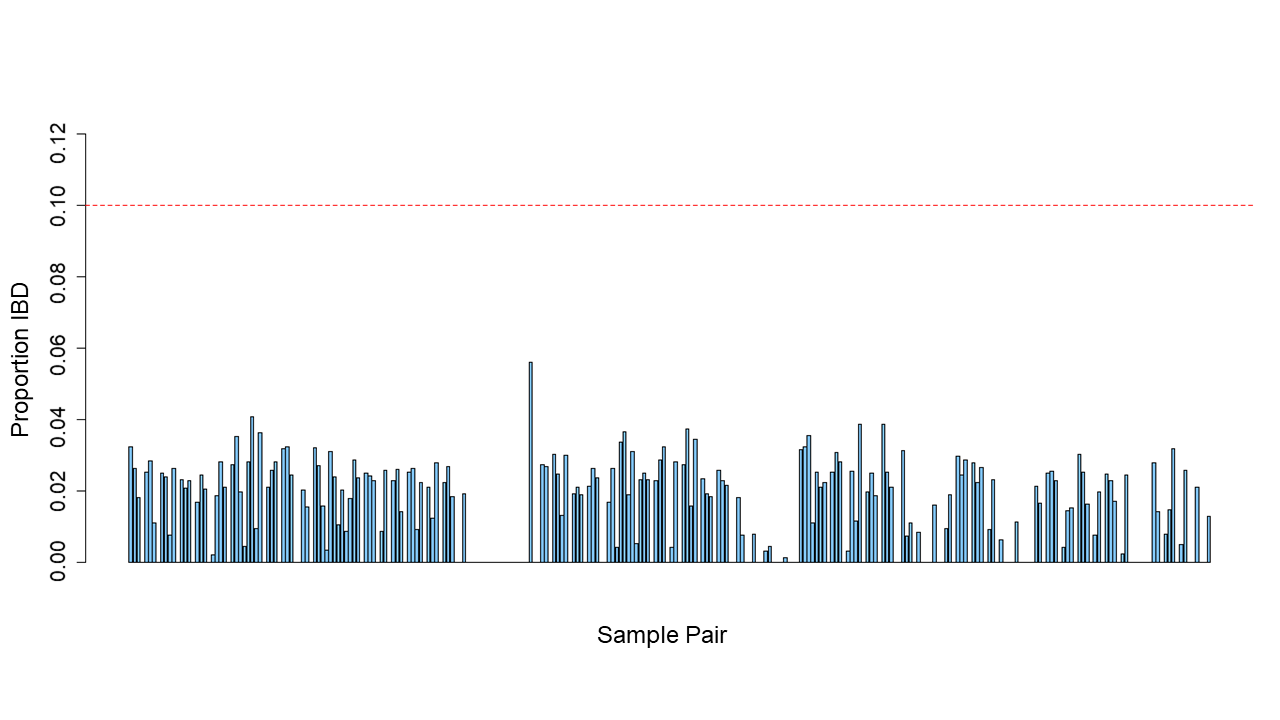

Supplement: Supplementary file 1 — Additional file 1. Supplementary Materials and Methods. [file 13023_2020_1648_MOESM1_ESM.docx]
